# Correction to “Quality of Life and Functioning Impairments Across Psychiatric Disorders in Adults Presenting for Outpatient Psychiatric Evaluation and Treatment”

**DOI:** 10.1176/rcp2.70030

**Published:** 2025-10-09

IsHak, W.W., Mirocha, J., Dang, J., Vanle, B., Metrikin, B., Tessema, K. and Danovitch, I. (2024), Quality of Life and Functioning Impairments Across Psychiatric Disorders in Adults Presenting for Outpatient Psychiatric Evaluation and Treatment. *Psych Res Clin Pract*, 6: 68–77. https://doi.org/10.1176/appi.prcp.20230064

In the article cited above, Alexander J. Steiner, Psy.D. has been added as the seventh author by agreement of all other authors. Due to an inadvertent error, Dr. Steiner had been omitted from the initial manuscript submission.

The corrected citation is shown below.

IsHak, W.W., Mirocha, J., Dang, J., Vanle, B., Metrikin, B., Tessema, K., Steiner A.J. and Danovitch, I. (2024), Quality of Life and Functioning Impairments Across Psychiatric Disorders in Adults Presenting for Outpatient Psychiatric Evaluation and Treatment. *Psych Res Clin Pract*, 6: 68–77. https://doi.org/10.1176/appi.prcp.20230064

We regret this error.

